# Duration of of Low-Temperature Storage, Clove Topping and Gibberellic Acid on Emergence, Yield and Yield Components of Garlic

**DOI:** 10.1155/2022/2998190

**Published:** 2022-03-24

**Authors:** Bizuayehu Desta, Kebede Woldetsadik, Wassu Mohammed, Netsanet Tena

**Affiliations:** ^1^School of Plant Sciences, College of Agriculture and Environmental Sciences, Haramaya University, Dire Dawa, Ethiopia; ^2^Department of Plant Sciences, College of Agriculture and Natural Resource Sciences, Debre Berhan University, Debre Berhan, Ethiopia

## Abstract

Dormancy of freshly harvested garlic cloves does not allow early emergence, and preplanting garlic clove treatment is critical for more than one cycle of production in a year. This field experiment was conducted to assess the effect of treating cloves on emergence, growth, and yield of “Tseday” variety during the main cropping season of 2014/2015 and off-season of 2015/16 at Haramaya University. The experiment was arranged in a factorial combination of four (cold stored at 7°C for the duration of 10, 20, and 30 days and stored at room temperature 21°C for 30 days as control), two (whole and topped clove), and four GA_3_ treatment at concentration of 0, 125, 250, and 375 mg/l and distilled water treatment as second control laid out in randomized complete block design with three replications. The three main factors (clove type, cold storage duration, and GA_3_) and growing season showed significant influence on phenology, growth, bulb yield and yield components, and all size categories of cloves, while GA_3_ showed nonsignificant effect on days to emergence of cloves. Clove type interacted with cold storage duration and GA_3_, and cold storage duration interacted with GA_3_ significantly to influence all characters of the variety. In addition, the growing season also interacted with clove type and GA_3_ to influence all categories of clove size, bulb diameter, average bulb weight, and total bulb yield. The three main factors (clove type × cold storage duration × GA_3_) interaction significantly influenced all characteristics of the variety. However, 30 days of cold-stored and topped cloves reduced dormancy period and days to maturity by 18.84 and 19.50 days, respectively, and increased total bulb yield by 70.32% as compared to the control treatment. In addition, this treatment combination significantly increased most of the growth and bulb yield components, while the number and weight of small-sized cloves were decreased. Hence, it can be concluded that 30 days of cold storage and topping of cloves without soaking under GA_3_ could be recommended to enhance early emergence, good vegetative growth, and total bulb yield of the garlic variety both under rain-fed and irrigated conditions.

## 1. Introduction

Garlic (*Allium sativum* L.) belongs to Alliaceae family, and it is believed to be originated in Central Asia (India, Afghanistan, West China, and Russia) and spread to other parts of the world through trade and colonization and used worldwide as a spice, functional food, and medicinal plant [1]. Garlic bulbs have a dormancy period of more than three months. Its sprouting and emergence are controlled mainly by temperature [2]. The early growth stage of garlic is suited by exposure of cloves to low temperature. Such exposure could be achieved using controlled temperature chambers such as refrigerators [3] or planting in a cool growing period, and this treatment is essential for proper development of shoot and good yield of bulb [4, 5].

The chilling requirement for improved bulbing in garlic can be supplemented by low-temperature treatment of mother bulbs prior to planting [6]. Siddique and Rabbani [6] reported that the treatment of mother bulbs at 6°C for 50 days before planting shortened the dormancy period and increased the bulb size and yield of garlic, particularly when the crop was planted late in the season.

Many studies have indicated that the application of growth promoter gibberellic acid can affect the growth and development of bulb crops and total bulb yield [7–9]. The treatment of seed bulbs (cloves) with gibberellic acid solution stimulated sprouting and bulbing and its development [10]. Ouzounidou et al. [11] also found that gibberellic acid promoted the total plant height in onion and garlic by 35% and 25% over the control, respectively. In addition, the number of leaves per plant and fresh and dry weight in both onion and garlic increased significantly under gibberellic acid.

Likewise, bulbs receiving cutting (topping) treatments sprouted earlier and uniformly than whole bulbs in shallot, *Allium wakegi,* and onion [12, 13]. This could be due to the removal of ABA; a sprout inhibiting substance existed in the removed portion [14].

One of the constraints of garlic production in Ethiopia is scarcity of planting materials since bulbs require the intermediate rest period and, thus, do not suit for immediate use. Hence, preplanting treatment such as low-temperature storage, clove topping, and GA_3_ for freshly harvested garlic clove seed would be imperative for easy sprouting and possibility of having two crop cycles per year. However, there is scarce information regarding the effects of these treatments on growth, yield, and yield components of garlic cultivars. Thus, the objective of this study was to determine the effectiveness of low-temperature storage, clove topping, and GA_3_ on growth, yield, and yield components of improved garlic variety, “Tseday.”

## 2. Materials and Methods

### 2.1. Description of the Experimental Site

The experiment was conducted at the experimental farm of Haramaya University (Raare, 505 km east of the capital, Addis Ababa, at 9° 26′ N latitude and 42° 03′ E longitude and an altitude of 2,022 masl) in eastern Ethiopia and done twice during rainy season and under off-season using irrigation in 2014/15 and 2015/16, respectively. Haramaya is situated in the semiarid tropical belt of eastern Ethiopia. The area has a bimodal rainfall distribution; the short rainy season stretches from March to May and the main rainy season from July to September. During the field experiment, the mean monthly minimum and maximum temperatures were 8.55/11.85°C and 24.83/25.6°C with 56.23/42.86% mean relative humidity during the rainy season (2014/15) and dry season (2015/16), respectively.

### 2.2. Treatments and Experimental Design

The treatment consisted of 2 × 4 × x 4 factorial combinations of clove types (whole and topped cloves), cold storage durations (10, 20, and 30 days) at 7°C plus one stored at room temperature and gibberellic acid concentrations ((0 mg/l or nonsoaked and soaked in distilled water), 125, 250, and 375 mg/l GA_3_).

Cloves were soaked in distilled water as the additional control treatment, but the results of statistical analyses showed nonsignificant difference with mean values of nonsoaked cloves for all characters; therefore, only nonsoaked cloves are considered as check/control treatment. The treatments were arranged in a randomized complete block design with three replications.

### 2.3. Planting and Agronomic Practices

Experimental plots were ploughed, thoroughly pulverized, and leveled, and ridges of about 20 cm high were prepared. The plot size was 3.6 m^2^ (1.8 m × 2 m). A distance of 1 m between the plots and 1.5 m between blocks was maintained. A garlic cultivar called “Tseday” was used for the experiment. The cloves (2.5–3.0 g) were planted on June 15, 2015, during the rainy season, and on October 15, 2016, during off-season according to the standard planting density of 10 × 30 cm with 20 plants per row. Fertilizer was applied at the rate of 105 N kg ha^−1^ and 92 P_2_O_5_ kg ha^−1^ in the form of DAP and urea with the equivalent amount of 200 and 150 kg ha^−1^, respectively, where all the recommended rate of DAP was applied at planting. Urea was applied in split, one-third during planting, one-third at active vegetative growth (three weeks after plant emergence), and the rest one-third six weeks after plant emergence as side dressing. Weeding, chemical sprays, and harvesting were done as per the recommendation made for the crop [15].

### 2.4. Data Collection

Ten sample plants from each plot were randomly tagged from the middle four rows, and data were recorded on the growth parameters of garlic. Growth parameters measured from the sample plants were plant height (cm), leaf length and width (cm), and shoot dry matter (g). Additionally, days to 50% emergence of shoots and 75% of maturity of plants were recorded. Yield components were measured after harvest from each treatment viz. neck diameter (cm), bulb length and diameter (cm), average bulb weight (g), clove number and average clove weight (in number and gram, respectively), total dry biomass (g), and clove size category (in number and gram). Clove size categories were determined as follows: very large (>2.50 g), large (2.0–2.50 g), medium (1.50–1.90 g), and small (1.0–1.49 g) [16].

Total bulb yield was calculated from total plants harvested from the central four rows of each plot and expressed on a hectare basis. To determine bulb dry matter, cloves from five randomly selected bulbs were chopped into small pieces with the help of stainless steel knife and mixed thoroughly, and the exact weight of each sample was determined and recorded as fresh weight. The samples were placed in paper bags and dried in an oven at 70°C until a constant weight was obtained. Each sample was immediately weighed using a digital sensitive balance and recorded as dry weight. Percent dry matter content for each sample was calculated by the following formula:

(1) $$\begin{array}{c} \text{DW}=\frac{\left[\left(\text{DW}+\text{CW}\right)-\text{CW}\right]}{\left[\left(\text{FW}+\text{CW}\right)-\text{CW}\right]}\;x\;100, \end{array}$$

where DW = dry weight, CW = container weight, and FW = fresh weight.

### 2.5. Data Analysis

Data obtained were subjected to analysis of variance (ANOVA) using the general linear model (GLM) of the SAS Statistical Package Version 9.2. Combined ANOVA over seasons was computed after homogeneity of the variances of the two seasons for all characters of the variety was tested by the F-test [17]. All significant pairs of treatment means were compared using Duncan's multiple range test (DMRT) at 5% level of significance.

## 3. Results and Discussion

### 3.1. Effect of Clove Treatments on Phenology and Growth of Garlic

The three main factors (clove type, cold storage duration, and GA_3_) and growing season had significant influence on phenology and growth of “*Tseday*,” while GA_3_ did not influence significantly the days to emergence of cloves. Clove type interacted with cold storage duration and GA_3_ and cold storage duration × GA_3_ interaction significantly influenced all phenology and growth, while growing season interacted with clove type and cold storage duration to influence days to emergence of cloves. All phenology and growth characteristics were significantly influenced by the interaction of clove type × cold storage duration × GA_3_ (Table 1).

#### 3.1.1. Phenology

Thirty days of cold-stored and topped cloves treated as control emerged about 18.84 days earlier than the control (non-cold-stored, non-topped, and non-GA_3_-treated cloves), and plant maturity was reduced by 19.5 days. However, cloves stored at ambient temperature, topped, and GA_3_-treated resulted in the longest duration for maturity. The application of GA_3_ at a rate of 250 and 375 mg/l on ambient temperature stored and non-topped cloves showed significantly earlier emergence and delayed maturity as compared to 0 and 125 mg/l GA_3_-treated cloves (Table 2).

Earlier emergence and maturity of cold-stored and topped cloves could be due to the earlier sprouting of cloves and subsequent faster sprout growth. Earlier sprouting by clove topping could be due to the removal of sprout inhibiting substances contained in the removed scale portion as suggested by Lin and Roberts [18] and Wang and Roberts [19] in Easter lily. The substance inhibiting sprouting in the bulbs of *Allium wakegi* has been proved to be abscisic acid [14]. Similarly, Yamazaki et al. [20] and Teaster et al. [21] also reported that the decrease in endogenous ABA content led to early sprouting. In agreement with this, Arifin et al. [12] reported that bulbs received cutting treatments sprouted earlier than whole bulbs in all accessions of shallot and *Allium wakegi*.

Solomina [22] and Takagi [2] reported the early emergence of seed cloves stored at 7°C and attributed this to the effect of low temperature in reducing the proportion of growth inhibitors and increase in growth-promoting hormones, especially gibberellins. Silva and Casali [23] also reported that cold storage for 30 and 40 days reduced the dormant period and increased field emergence. In addition, Langens-Gerrits et al. [24] observed that shoot emergence from dormant bulblets of *Lilium speciosum* occurred more quickly with greater uniformity after a longer chilling (6 weeks) duration at 5°C.

Several researchers have reported the early maturity of garlic and onions after a preplanting low-temperature treatment [25, 26]. The findings agree with the research of Aura [27], Butt [28], Palilov [29], and Khokhar et al. [26] on onions; under low storage temperatures (0–7°C), the vegetative cycle is reduced and bulbing is accelerated, while under high temperatures (18–30°C), both bulbing and ripening are delayed.

Rahim and Fordham [30] also showed that garlic cloves treated with cold temperatures at 5 or 10°C for 15 to 30 days before planting had accelerated maturity of bulbs relative to those stored at 15 and 20°C. This is due to the ability of long cold storage duration to effectively break dormancy followed by early emergence rate for easy stand establishment and initiation of bulbing and thereby ending its physiological activity of growth and development than non-cold and non-topped treated cloves [12, 25, 31, 32].

The earlier sprouting and longest duration of maturity by GA_3_ could be due to rapid stimulation of sprouting and vigorous vegetative growth. In line with this, Rahman et al. [33] reported that the sprouting percentage of *Allium sativum* increased in bulbs treated with GA_3_, where the highest percentage of germination was achieved at the concentration of 250 ppm. On the other hand, Rahman et al. [33] indicated that GA_3_ treatment at 250 ppm delayed bulb maturity compared with those treated with lower concentrations and nontreated ones. GA_3_ delays maturity because of its physiological effects in the stimulation of vegetative growth [33–35].

#### 3.1.2. Growth Characteristics

Storage of cloves at cold temperature for 30 days coupled with topping and no GA_3_ application resulted in the highest plant height, leaf length and width, and shoot dry mass. However, cloves stored at ambient temperature, topped, and treated with GA_3_ gave the smallest values of these parameters followed by 10 days of cold-stored, topped, and GA_3_-treated cloves. A significant increase in plant height, leaf length and width, and shoot dry mass was observed by the application of GA_3_ at a rate of 250 and 375 mg/l on ambient temperature stored and non-topped cloves as compared to 0 and 125 mg/l GA_3_-treated cloves (Table 3).

The increase in growth characters by GA_3_ could possibly be due to early sprouting of cloves and proper growth of vegetative parts by GA_3_ application that lack sufficient amount of cold temperature exposure [11, 36]. In accordance with this, Ouzounidou et al. [11] reported that 100 ppm GA_3_ application after three weeks of germination promoted elongation of onion and garlic plants by 35% and 25%, respectively, over the control. On the contrary, Rahman et al. [37] reported that plant height decreased with increased concentration of GA_3_ to 200 ppm spry application. Rahman et al. [33] reported a difference result that showed nonsignificant effect of GA_3_ on plant height and enhanced initiation and development of garlic leaf growth.

The significant increase in growth characters by clove topping and cold storage could possibly be due to early sprouting of cloves, which enabled proper vegetative growth. Such an increase in vegetative growth by topping [38, 39] and cool temperature [4, 5] resulted in increased leaf growth of the plant. In agreement with this, Dutcher and Powell [40] and Singha and Powell [41], who used apple *(Malus domestica* Borkh cv Northern Spy) buds, found that ABA inhibited bud break and shoot elongation.

Satin and Lopez [25] indicated that bulbs that received low-temperature storage had increased plant growth compared with nontreated counterparts. Lucidos et al. [42] also showed that exposure of bulbs to 4°C for 65 days promoted stem elongation in *Lilium hansonii*. In addition, Kurtar and Ayan [43] reported that exposure of tulips to low temperature increased the production of gibberellins and auxins, which were necessary for stalk elongation.

### 3.2. Effect of Clove Treatments on Yield and Yield Components of Garlic

The growing season and the three main factors significantly influenced bulb yield and yield components of “*Tseday*” variety. Clove type interacted with cold storage duration and GA_3_ to influence all bulb yield and yield components of the variety. Interaction of growing season × clove type and growing season × GA_3_ significantly influenced bulb diameter, average bulb weight, and total bulb yield. In addition, the interaction of growing season × cold storage duration significantly influenced bulb diameter and average clove weight. All bulb yield and yield components of the variety were significantly influenced by the interaction of clove type × cold storage duration × GA_3_, while bulb diameter, average bulb weight, and total bulb yield were significantly influenced by the interaction of season × clove type × GA_3_ (Table 4).

#### 3.2.1. Bulb Characteristics

**Figure 1:**
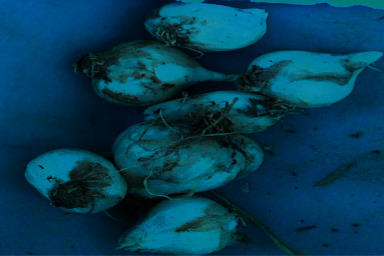
Topped clove, 30 days of cold storage, and 0 mg/l GA_3_ (2 months after planting).

**Figure 2:**
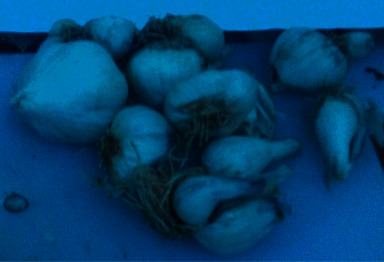
Topped clove, ambient temperature stored, and 375 mg/l GA_3_ (after harvesting).

**Figure 3:**
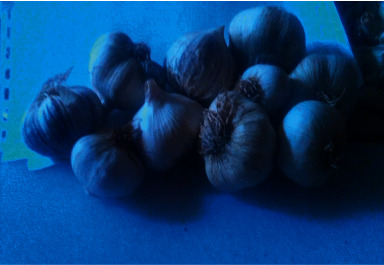
Topped clove, 30 days of cold storage, and 0 mg/l GA_3_ (after harvesting).

**Figure 4:**
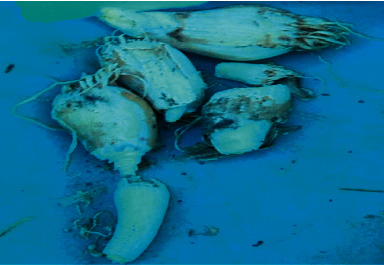
Whole clove, 30 days of cold storage, and 0 mg/l GA_3_ (2 months after planting).

**Figure 5:**
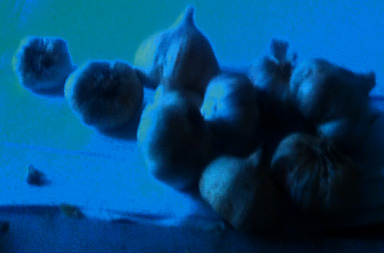
Whole clove, 30 days of cold storage, and 0 mg/l GA_3_ (after harvesting).

The highest neck diameter, bulb length and diameter, and average bulb weight were recorded from 30 days of cold-stored and topped cloves treated with no GA_3_. However, cloves stored at ambient temperature, topped, and treated by GA_3_ gave the smallest values of these parameters followed by 10 days of cold-stored, topped, and GA_3_-treated cloves (Figures 1–3 and Table 5). These bulb characters were also significantly increased by treating with GA_3_ at a rate of 250 and 375 mg/l on ambient temperature stored and non-topped cloves as compared to 0 and 125 mg/l GA_3_-treated cloves (Table 5). In addition, significantly higher values of these characters were also observed from 30 days of cold-stored and topped cloves with no GA_3_ as compared to 30 days of cold-stored and non-topped cloves with no GA_3_ treatment (Figures 1, 3–5 and Table 5).

The increase in bulb size by low temperature could be due to the increase in auxin and GA_3_ production that in turn stimulates clove differentiation and development of larger bulbs [44]. In line with this, several authors reported that low temperature before planting the cloves increased the bulb size of garlic that eventually reflected in bulb length and diameter [6, 25, 45, 46].

The increase in bulb characters due to cold storage, topping, and GA_3_ could be the results of early emergence and an increase in the vegetative growth status before moisture and nutrient reserves dwindle. The vigorous vegetative growth accumulates more net photosynthates, which are then translocated to bulbs forming larger bulbs.

In agreement with the present result, Rahman et al. [33] and Ade-Ademilua et al. [5] reported that the pretreatment of garlic cloves with cold temperature and GA_3_ could have helped the plants to improve their ability to use available growth resources, moisture, and nutrients.

#### 3.2.2. Clove Characteristics

Preplant cold treatment of cloves for 30 days and topping with no GA_3_ application resulted in the highest number of cloves per bulb, while average clove weight was significantly higher for 30 and 20 days of cold-stored and topped cloves treated with no GA_3_. Clove number and average clove weight were significantly increased by the application of GA_3_ at a rate of 250 and 375 mg/l on ambient temperature stored and non-topped cloves as compared to 0 and125 mg/l GA_3_-treated cloves (Table 6).

The increase in clove number and weight due to GA_3_ [10], cold storage [25, 44, 45], and clove topping might be due to the earliest sprouting and the subsequent development of large bulbs caused by vigorous vegetative growth [47]. On the contrary, Rahim and Fordham [48] and Youssef [46] reported that preplant cold treatment of cloves reduced the number of cloves per bulb.

#### 3.2.3. Bulb Dry Matter Percentage and Total Dry Biomass

Cloves cold stored for 30 days, topped, and with no GA_3_ treatment resulted in the highest bulb dry matter percentage and total dry biomass. However, cloves stored at ambient temperature, topped, and treated by GA_3_ gave the smallest values of these parameters.

The application of GA_3_ at a rate of 250 and 375 mg/l on ambient temperature stored and non-topped cloves showed a significant increase in bulb dry matter percentage and total dry biomass as compared to 0 and 125 mg/l GA_3_-treated cloves (Table 7).

The higher bulb dry matter percentage and total dry biomass by low temperature could be due to the vigorous vegetative growth that accumulates more net photosynthates, which are then translocated to bulbs and forming a higher dry matter accumulation in the bulbs. In line with this, Qaryouti and Kasrawi [47] reported that higher dry weight of bulbs was produced from cloves stored at 0 or 10°C than those stored at room temperature (17–22°C) as a result of the vigorous vegetative growth. De Klerk [49] also reported lower values of dry weight of lily bulbs exposed to 15°C than 4°C.

The increase in total dry biomass and bulb dry matter percentage by GA_3_ could possibly be due to the early breakdown of clove dormancy and its effect on cell division and elongation, which enhanced shoot growth. In addition, Daykin et al. [34], Hisamatsu et al. [35], and Rahman et al. [33] reported that the application of GA_3_ stimulated vegetative growth, which could increase the dry matter content of bulbs. Rahman et al. [37] also reported that the spray application of GA_3_ significantly increased total dry biomass.

The increase in total dry biomass and bulb dry matter percentage by cold treatment [4, 5] and topping [38, 39] might be due to earlier sprouting and the subsequent vigorous vegetative growth of the plant, which increased dry mass of the plant.

#### 3.2.4. Total Bulb Yield

The highest bulb yield was recorded from 30 days of cold-stored and topped cloves treated with no GA_3_ treatment followed by 20 days of cold-stored and topped cloves treated with no GA_3_ application. However, cloves stored at ambient temperature, topped, and treated by GA_3_ gave the smallest total bulb yield followed by 10 days of cold-stored, topped, and GA_3_-treated cloves (Figures 1–3 and Table 7).

**Figure 6:**
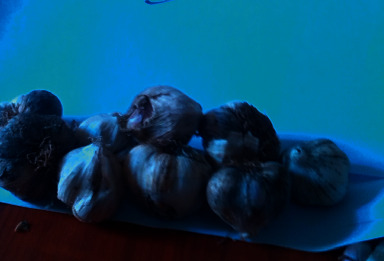
Topped clove, 30 days of cold storage, and 125 mg/l GA_3_ (after harvesting).

**Figure 7:**
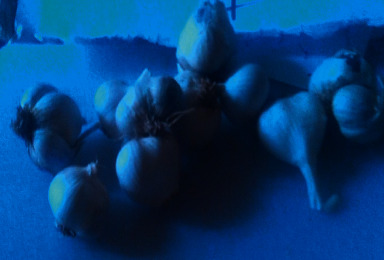
Topped clove, 30 days of cold storage, and 250 mg/l GA_3_ (after harvesting).

**Figure 8:**
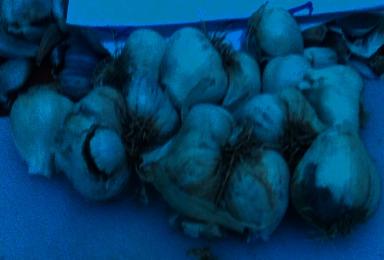
Topped clove, 30 days of cold storage, and 375 mg/l GA_3_ (after harvesting).

A significant increase in total bulb yield was observed by the application of GA_3_ at a rate of 250 and 375 mg/l on ambient temperature stored and non-topped cloves compared with 0 and 125 mg/l GA_3_-treated cloves (Table 7). A significantly higher value of total bulb yield was also recorded from 30 days of cold-stored and topped cloves with no GA_3_ treatment as compared to 30 days of cold-stored and non-topped cloves with no GA_3_ treatment (Figures 1, 3–5 and Table 7). In addition, a significantly higher value of bulb yield was observed from 30 days of cold-stored and topped cloves with no GA_3_ treatment as compared to 30 days of cold-stored and topped cloves with all rates of GA_3_ treatment (Figures 1, 3, 6–8 and Table 7). The increase in total bulb yield by GA_3_ could be attributed to the positive impact of optimum concentrations of GA_3_ on vegetative growth that resulted in higher bulb yield. In agreement with this, Rahman et al. [33] reported that the application of GA_3_ stimulated vegetative growth, which could increase the bulb yield of garlic.

The production of higher yields from seed cloves stored at 7°C and topped may be due to the development of higher vegetative growth, measured as plant height and leaf number, before initiation of bulbing as compared to lower vegetative growth obtained from seed cloves stored at room temperature. Plants need to achieve adequate growth before bulbing commences, so that the foliage can produce large bulbs and high yields [50, 51]. Satin and Lopez [25] reported that total bulb yield was higher when cloves were stored at 0°C and 7°C for 30 days than cloves stored at 25°C for 30 days. Bhuiya et al. [4] also reported that low-temperature exposure is essential for proper development of shoot and good yield of bulb.

In addition, Bandara et al. [52] found that increasing the chilling treatment (4°C) period up to 45 days prior to field planting increased the bulb yield, while a further increase in the treatment period reduced the yield. However, there are many contradictions on the effect of the preplanting low-temperature treatment on garlic yield; some researchers have reported increases [53], while others have observed no significant differences [54]; still, others have reported depressive effects [55].

### 3.3. Effect of Clove Treatments on Clove Sizes of Garlic

The growing season, three main factors, the interaction of clove type with cold storage duration and GA_3_, and the interaction of cold storage duration and GA_3_ significantly influenced all categories of the clove size of the variety. In addition, the interaction of growing season and clove type significantly influenced cloves under large (in number basis), and very large and small size (in weight basis) categories. The growing season also interacted with cold storage duration to affect significantly large and very large cloves, while it interacted with GA_3_ and with clove type × GA_3_ significantly influenced cloves under large size category on weight basis. All categories of the clove size of the variety were significantly influenced by the interaction of clove type *×* cold storage duration × GA_3_ (Table 8).

Planting of 30 days of cold-stored and topped cloves treated with no GA_3_ showed the smallest number and weight of small-sized cloves per bulb. On the other hand, the same treatment resulted in the highest number and weight of medium-sized, large-sized, and very large-sized cloves per bulb. Cloves stored at ambient temperature, topped, and GA_3_-treated recorded the highest number and weight of small-sized cloves and the lowest number and weight of medium-sized, large-sized, and very large-sized cloves per bulb (Tables 9, 10).

Gibberellic acid treatment at a rate of 250 and 375 mg/l on ambient temperature stored and non-topped cloves showed a significant decrease in number and weight of small-sized cloves per bulb and an increase in number and weight of medium-sized, large-sized, cloves per bulb as compared to 0 and 125 mg/l GA_3_-treated cloves. GA_3_ application at a rate of 250 mg/l on 20 days of cold-stored and non-topped cloves showed a comparable weight of small-sized cloves, number and weight of large-sized cloves, number of medium-sized cloves, and weight of very large-sized cloves with 30 days of cold-stored and non-topped cloves treated with GA_3_ (Tables 9, 10). Similarly, this treatment showed a comparable weight of medium-sized cloves with 30 days of cold-stored and non-topped cloves treated with 125 and 250 mg/l GA_3_ application. The number and weight of medium-sized, large-sized, and very large-sized clove increment by GA_3_ application could be due to early sprouting and bulbing and its development. In agreement with this, Moon and Lee [10] reported that preplant treatment of cloves with GA_3_ produced positive impact on the number and weight of cloves.

The consistent increase in number and weight of medium-sized, large-sized, and very large-sized cloves and decreasing number and weight of small-sized cloves in response to planting of 30 days of cold-stored, topped, and no GA_3_-treated cloves could be due to the effect of clove topping, low temperature, and GA_3_ in early sprouting and bulbing. Clove topping treatment results in earlier and uniform sprouting that favors bulbing [12, 13]. This is due to the removal of ABA, and a sprout inhibiting substance existed in the removed portion [14]. Low temperature also increases auxin and GA_3_ production that in turn stimulates clove differentiation and development of larger bulbs [44]. Youssef [46] also reported that decreasing temperature (10°C) and increasing storage duration up to 30 days increased clove weight significantly, while the decreased storage duration leads to reduced clove size. In addition, Bandara et al. [52] and Manjula et al. [56] also reported that preplant chilling treatments of cloves significantly increased clove size.

## 4. Conclusion

For cloves stored at ambient temperature (0 day) and non-topped, gibberellic acid application at a rate of 250 and 375 mg/l significantly reduced dormancy period by 5.84 days while increasing days to maturity by 2.84 days and yield by 9.72% compared with the control. On the other hand, for topped cloves stored at 0, 10, 20, and 30 days of cold storage, GA_3_ treatment significantly delayed days to maturity and decreased yield of garlic, while it did not show a significant influence on days to emergence as compared to the controls. The 30 days of cold-stored and topped cloves with no GA_3_ treatment significantly reduced dormancy period and days to maturity while increasing the yield of garlic as compared to the controls. In addition, this treatment significantly increased most of the growth and bulb yield components, while the number and weight of small-sized cloves were decreased. Thus, the combination of cold storage for 30 days and topping without soaking under GA_3_ could be used to treat fresh garlic cloves for early emergence (19.16 days), good vegetative growth, and higher yield (13.66 t/ha) both under rain-fed and irrigated conditions.

## Figures and Tables

**Table 1:** Mean square for the effect of cold storage duration (DLTS), clove topping (CT), gibberellic acid (GA_3_), and season on days to emergence and maturity, plant height, leaf length and width, and shoot dry matter of garlic.

Source of variation	DAE	DTM	PLHT (cm)	LL (cm)	LW (cm)	SDM
Rep (2)	4.7375^ns^	51.2791^*∗∗*^	50.0415^*∗∗*^	14.4561^*∗∗*^	0.0602^*∗∗*^	0.1136^*∗∗*^
Sn (1)	1316.0166^*∗∗*^	3580.5375^*∗∗*^	1140.0419^*∗∗*^	1232.1914^*∗∗*^	0.2559^*∗∗*^	21.5303^*∗∗*^
CT (1)	799.3500^*∗∗*^	3352.5375^*∗∗*^	2342.2569^*∗∗*^	1696.3345^*∗∗*^	4.0352^*∗∗*^	23.9595^*∗∗*^
DLTS (3)	1411.5111^*∗∗*^	2022.9152^*∗∗*^	1759.2924^*∗∗*^	1310.4509^*∗∗*^	3.8462^*∗∗*^	16.0165^*∗∗*^
GA_3_ (4)	3.2458^ns^	2035.0833^*∗∗*^	1568.7495^*∗∗*^	1167.9849^*∗∗*^	2.7001^*∗∗*^	13.5002^*∗∗*^
CT *×* DLTS (3)	46.8611^*∗∗*^	15.8152^*∗∗*^	26.9672^*∗∗*^	22.5131^*∗∗*^	0.0206^*∗∗*^	0.1001^*∗∗*^
CT *×* GA_3_ (4)	17.9541^*∗∗*^	1655.6833^*∗∗*^	1858.5766^*∗∗*^	1379.3881^*∗∗*^	3.1207^*∗∗*^	16.8989^*∗∗*^
DLTS *×* GA_3_ (12)	7.6847^*∗∗*^	11.8666^*∗∗*^	32.4567^*∗∗*^	25.6409^*∗∗*^	0.0495^*∗∗*^	0.3184^*∗∗*^
Sn *×* CT (1)	13.0666^*∗*^	1.8375^ns^	2.4444^ns^	6.0049^ns^	0.0004^ns^	0.0374^ns^
Sn *×* DLTS (3)	17.6611^*∗∗*^	2.6819^ns^	0.3706^ns^	0.3204^ns^	0.00009^ns^	0.0269^ns^
Sn *×* GA_3_ (4)	0.0791^ns^	0.9541^ns^	0.5038^ns^	0.6322^ns^	0.00002^ns^	0.0207^ns^
CT *×* DLTS *×* GA_3_ (12)	11.0208^*∗∗*^	12.9333^*∗∗*^	9.6856^*∗∗*^	8.2097^*∗∗*^	0.0153^*∗∗*^	0.0685^*∗∗*^
Sn *×* DLTS *×* GA_3_ (12)	0.0569^ns^	0.0152^ns^	0.0512^ns^	0.0968^ns^	0.00007^ns^	0.0004^ns^
Sn *×* CT *×* DLTS (3)	1.6666^ns^	0.6486^ns^	1.3138^ns^	2.0167^ns^	0.00006^ns^	0.0001^ns^
Sn *×* CT *×* GA_3_ (4)	0.2541^ns^	1.1708^ns^	1.9274^ns^	2.0642^ns^	0.0001^ns^	0.0247^ns^
Sn *×* CT *×* DLTS *×* GA_3_ (12)	0.4375^ns^	0.0097^ns^	0.0982^ns^	0.1180^ns^	0.00003^ns^	0.0001^ns^
Error (158)	2.7332	2.8023	2.6927	1.956	0.0034	0.0161
CV (%)	6.02	1.1	3.14	3.63	3.35	3.87

ns, ^*∗*^and ^*∗∗*^nonsignificant, significant at *P* < 0.05 and *P* < 0.01, respectively.

**Table 2:** Interaction effects of clove topping, cold storage duration, and GA_3_ on days to emergence and maturity.

Storage	Topping	Whole clove		Topped clove	
	GA_3_ (mg/l)	DE	DM	DE	DM
Ambient temperature	0	38.00^a^	153.66^f^	30.50^bc^	150.66^g^
	125	37.33^a^	153.66^f^	30.50^bc^	168.83^a^
	250	32.16^b^	156.50^e^	32.16^b^	168.83^a^
	375	32.16^b^	156.50^e^	32.16^b^	168.83^a^
Cold storage (days)					

10	0	30.66^bc^	150.66^g^	28.50^de^	144.16^i^
	125	30.33^b-d^	150.66^g^	29.00^cd^	165.83^b^
	250	29.00^cd^	150.66^g^	29.00^cd^	165.83^b^
	375	29.00^cd^	150.66^g^	29.16^cd^	165.83^b^

20	0	26.66^ef^	144.33^i^	22.50^g^	137.83^l^
	125	26.66^ef^	144.33^i^	22.50^g^	163.00^c^
	250	26.16^f^	147.33^h^	22.16^gh^	163.00^c^
	375	26.66^ef^	144.66^i^	23.00^g^	163.00^c^

30	0	23.83^g^	139.50^kl^	19.16^i^	134.16^m^
	125	26.00^f^	140.50^jk^	20.50^hi^	159.33^d^
	250	26.00^f^	141.50^j^	20.50^hi^	159.33^d^
	375	26.00^f^	141.50^j^	20.50^hi^	159.33^d^

Overall mean	Topping	29.16	147.91	25.74	158.61
	Ambient	34.91	155.08	31.33	164.28
	Cold storage	27.25	145.52	23.87	156.72
	Nonsoaked	29.78	147.04	25.16	141.70
	GA_3_ soaked	28.95	148.20	25.93	164.25

Means with different letter(s) in columns have significant differences according to DMRT at 5% probability level, and DE = days to emergence and DM = days to maturity.

**Table 3:** Interaction effects of clove topping, cold storage duration, and gibberellic acid on leaf length and width, plant height, and shoot dry mass.

Storage	Topping	Whole clove				Topped clove			
	GA_3_ (mg/l)	LL (cm)	LW (cm)	PH (cm)	SDM (g)	LL (cm)	LW (cm)	PH (cm)	SDM (g)
Ambient temperature	0	34.98^h^	1.56^i^	48.11^h^	2.75^k^	40.03^f^	1.77^fg^	54.03^f^	3.41^fg^
	125	35.02^h^	1.57^i^	48.15^h^	2.92^j^	24.17^l^	0.99^m^	35.48^l^	1.78^o^
	250	37.53^g^	1.67^h^	51.07^g^	3.17^i^	24.09^l^	0.98^m^	35.38^l^	1.76^o^
	375	37.54^g^	1.68^h^	51.08^g^	3.18^i^	23.76^l^	0.99^m^	35.15^l^	1.76^o^
Cold storage (days)									

10	0	38.28^g^	1.70^h^	51.95^g^	3.26^hi^	42.94^e^	1.96^e^	57.42^e^	3.81^e^
	125	40.03^f^	1.77^fg^	54.02^f^	3.41^f-h^	26.96^k^	1.24^l^	38.79^k^	2.03^n^
	250	40.19^f^	1.78^f^	54.22^f^	3.43^f^	26.97^k^	1.21^l^	38.79^k^	2.03^n^
	375	40.20^f^	1.78^f^	54.23^f^	3.44^f^	26.97^k^	1.22^l^	38.80^k^	2.04^n^

20	0	42.98^e^	1.96^e^	57.47^e^	3.82^e^	48.18^b^	2.27^b^	63.51^b^	4.44^b^
	125	42.97^e^	1.97^e^	57.46^e^	3.82^e^	29.71^j^	1.37^k^	41.93^j^	2.28^m^
	250	44.17^de^	2.05^d^	58.90^de^	4.00^d^	29.73^j^	1.35^k^	41.95^j^	2.27^m^
	375	42.99^e^	1.97^e^	57.47^e^	3.83^e^	29.61^j^	1.34^k^	41.85^j^	2.26^m^

30	0	46.25^c^	2.17^c^	61.27^c^	4.25^c^	56.81^a^	2.58^a^	72.70^a^	5.04^a^
	125	45.43^cd^	2.14^c^	60.37^cd^	4.18^c^	32.45^i^	1.46^j^	45.20^i^	2.51^l^
	250	45.42^cd^	2.13^c^	60.28^cd^	4.17^c^	32.43^i^	1.47^j^	45.18^i^	2.52^l^
	375	45.46^cd^	2.14^c^	60.33^cd^	4.19^c^	32.41^i^	1.47^j^	45.15^i^	2.51^l^

Overall mean	Topping	41.21	1.88	55.40	3.61	32.95	1.48	45.71	2.65
	Ambient	36.27	1.62	49.60	3.00	28.01	1.18	40.01	2.18
	Cold storage	42.86	1.96	57.33	3.82	34.59	1.58	47.60	2.81
	Nonsoaked	40.62	1.85	54.70	3.52	46.99	2.14	61.91	4.17
	GA_3_ soaked	41.41	1.89	55.63	3.64	28.27	1.26	40.30	2.14

Means with different letter(s) in columns have significant differences according to DMRT at 5% probability level, and LL (cm) = leaf length in centimeter, LW = leaf width, PH = plant height, and SDM = shoot dry mass.

**Table 4:** Mean square for the effect of cold storage duration (DLTS), clove topping (CT), gibberellic acid (GA_3_), and season on clove number per bulb, average clove weight, average bulb weight, bulb dry matter percentage, total bulb yield, neck diameter, bulb length, diameter, and total dry biomass of garlic.

Source of variation	CLNPB	ACW	ABW	BDP	TBY	ND (cm)	BL	BD	TDB
Rep (2)	2.6305^*∗∗*^	0.0744^*∗∗*^	30.9063^*∗∗*^	24.1559^*∗∗*^	3.7346^*∗∗*^	0.01074^*∗∗*^	0.0876^*∗∗*^	1.9194^*∗∗*^	4.3936^*∗∗*^
Sn (1)	28.7110^*∗∗*^	0.0510^*∗∗*^	746.1252^*∗∗*^	492.0570^*∗∗*^	96.0596^*∗∗*^	2.08702^*∗∗*^	12.9360^*∗∗*^	11.0834^*∗∗*^	358.3713^*∗∗*^
CT (1)	26.6067^*∗∗*^	2.9173^*∗∗*^	1546.3757^*∗∗*^	355.7078^*∗∗*^	189.0729^*∗∗*^	0.34527^*∗∗*^	13.0803^*∗∗*^	19.0482^*∗∗*^	394.8893^*∗∗*^
DLTS (3)	25.2600^*∗∗*^	0.7188^*∗∗*^	828.9191^*∗∗*^	284.9727^*∗∗*^	93.0085^*∗∗*^	0.24518^*∗∗*^	10.8793^*∗∗*^	15.7129^*∗∗*^	341.6285^*∗∗*^
GA_3_ (4)	22.1964^*∗∗*^	0.9411^*∗∗*^	858.4323^*∗∗*^	242.9432^*∗∗*^	97.3249^*∗∗*^	0.21279^*∗∗*^	9.3321^*∗∗*^	13.4052^*∗∗*^	296.8911^*∗∗*^
CT × DLTS (3)	0.8397^*∗∗*^	0.0181^*∗∗*^	9.2643^*∗∗*^	1.7948^*∗∗*^	0.8825^*∗∗*^	0.00372^*∗∗*^	0.0706^*∗∗*^	0.1124^*∗∗*^	2.2605^*∗∗*^
CT × GA_3_ (4)	26.5302^*∗∗*^	1.0811^*∗∗*^	996.9291^*∗∗*^	271.5802^*∗∗*^	112.7276^*∗∗*^	0.25592^*∗∗*^	10.2841^*∗∗*^	14.8878^*∗∗*^	345.4027^*∗∗*^
DLTS × GA_3_ (12)	0.8267^*∗∗*^	0.0042^*∗∗*^	17.7893^*∗∗*^	5.6298^*∗∗*^	1.7253^*∗∗*^	0.00350^*∗∗*^	0.2291^*∗∗*^	0.3342^*∗∗*^	10.6196^*∗∗*^
Sn × CT (1)	0.0182^ns^	0.0015^ns^	33.1448^*∗∗*^	0.0100^ns^	7.5776^*∗∗*^	0.00031^ns^	0.0296^ns^	0.1219^*∗∗*^	0.7627^*∗*^
Sn × DLTS (3)	0.0600^ns^	0.0032^*∗*^	0.7235^ns^	0.0692^ns^	0.2263^ns^	0.00013^ns^	0.0238^ns^	0.1014^*∗∗*^	0.0190^ns^
Sn × GA_3_ (4)	0.0474^ns^	0.0006^ns^	8.5163^*∗∗*^	0.0125^ns^	1.8416^*∗∗*^	0.00005^ns^	0.0219^ns^	0.0944^*∗∗*^	0.1511^ns^
CT × DLTS × GA_3_ (12)	0.5242^*∗∗*^	0.0040^*∗∗*^	5.4572^*∗∗*^	1.7699^*∗∗*^	0.4474^*∗∗*^	0.00109^*∗*^	0.0741^*∗∗*^	0.1042^*∗∗*^	4.0534^*∗∗*^
Sn × DLTS × GA_3_ (12)	0.0036^ns^	0.00006^ns^	0.1090^ns^	0.0229^ns^	0.0361^ns^	0.00003^ns^	0.0005^ns^	0.0021^ns^	0.0150^ns^
Sn × CT × DLTS (3)	0.0001^ns^	0.0008^ns^	0.2304^ns^	0.0100^ns^	0.1696^ns^	0.00010^ns^	0.0001^ns^	0.0007^ns^	0.0686^ns^
Sn × CT × GA_3_ (4)	0.0530^ns^	0.0013^ns^	7.8289^*∗∗*^	0.0167^ns^	1.7197^*∗∗*^	0.00009^ns^	0.0228^ns^	0.0985^*∗∗*^	0.1183^ns^
Sn × CT × DLTS × GA_3_ (12)	0.0023^ns^	0.00004^ns^	0.0253^ns^	0.0164^ns^	0.0055^ns^	0.00008^ns^	0.0001^ns^	0.0007^ns^	0.0064^ns^
Error (158)	0.0664	0.001	0.7679	0.2899	0.09	0.0005	0.0109	0.0162	0.1876
CV (%)	3.06	1.31	3.52	1.82	3.37	2.14	3.45	3.45	3.65

ns, ^*∗*^and ^*∗∗*^nonsignificant, significant at *P* < 0.05 and *P* < 0.01, respectively.

**Table 5:** Neck diameter, bulb length and diameter, and average bulb weight of garlic cloves as influenced by the interaction effects of clove topping, cold storage duration, and gibberellic acid.

Storage	Topping	Whole clove				Topped clove			
	GA_3_ (mg/l)	BL(cm)	BD (cm)	ABW (g)	ND (cm)	BL(cm)	BD (cm)	ABW (g)	ND (cm)
Ambient temperature	0	2.69^j^	3.26^i^	22.14^i^	1.01^i^	3.14^h^	3.80^g^	26.31^fg^	1.09^fg^
	125	2.71^j^	3.29^i^	22.45^i^	1.02^i^	1.81^n^	2.20^m^	12.98^m^	0.85^m^
	250	2.91^i^	3.53^h^	24.36^h^	1.05^h^	1.79^n^	2.18^m^	12.83^m^	0.86^m^
	375	2.92^i^	3.54^h^	24.38^h^	1.05^h^	1.79^n^	2.19^m^	12.96^m^	0.85^m^
Cold storage (days)									

10	0	3.07^h^	3.72^g^	25.31^gh^	1.06^gh^	3.40^g^	4.11^f^	28.76^e^	1.12^e^
	125	3.15^h^	3.82^g^	26.47^f^	1.08^fg^	2.01^m^	2.44^l^	14.89^l^	0.90^l^
	250	3.15^h^	3.84^g^	26.58^f^	1.09^f^	2.02^m^	2.45^l^	14.91^l^	0.90^l^
	375	3.16^h^	3.84^g^	26.62^f^	1.09^f^	2.02^m^	2.46^l^	14.94^l^	0.91^l^

20	0	3.38^g^	4.10^f^	28.95^e^	1.12^e^	3.94^b^	4.76^b^	33.00^b^	1.19^b^
	125	3.39^g^	4.11^f^	28.99^e^	1.12^e^	2.27^l^	2.75^k^	16.95^k^	0.94^k^
	250	3.54^f^	4.29^e^	30.03^d^	1.14^de^	2.27^l^	2.77^k^	16.97^k^	0.94^k^
	375	3.38^g^	4.10^f^	28.98^e^	1.12^e^	2.28^l^	2.78^k^	16.96^k^	0.95^k^

30	0	3.81^cd^	4.61^c^	31.77^c^	1.17^bc^	4.64^a^	5.60^a^	39.43^a^	1.29^a^
	125	3.69^e^	4.47^d^	30.90^cd^	1.16^cd^	2.48^k^	3.00^j^	18.91^j^	0.98^j^
	250	3.68^e^	4.46^d^	31.06^c^	1.16^cd^	2.49^k^	3.02^j^	18.92^j^	0.98^j^
	375	3.70^de^	4.48^cd^	31.09^c^	1.16^cd^	2.48^k^	3.02^j^	18.95^j^	0.99^j^

Overall mean	Topping	3.27	3.96	27.50	1.10	2.55	3.09	19.92	0.98
	Ambient	2.81	3.40	23.33	1.03	2.13	2.59	16.27	0.91
	Cold storage	3.42	4.15	28.89	1.12	2.69	3.26	21.13	1.01
	Nonsoaked	3.24	3.92	27.04	1.09	3.78	4.57	31.87	1.17
	GA_3_ soaked	3.28	3.98	27.66	1.10	2.14	2.60	15.93	0.92

Means with different letter(s) in columns have significant differences according to DMRT at 5% probability level, and ND = neck diameter, BL = bulb length, BW = bulb diameter, and ABW = average bulb weight.

**Table 6:** Clove number and average clove weight of garlic cloves as influenced by the interaction effects of clove topping, cold storage duration, and gibberellic acid.

Storage	Topping	Whole clove		Topped clove	
	GA_3_ (mg/l)	CN	ACW (g)	CN	ACW (g)
Ambient temperature	0	7.89^g^	2.43^l^	8.53^e^	2.60^gh^
	125	7.89^g^	2.44^l^	6.61^m-o^	2.11^pq^
	250	8.20^f^	2.52^k^	6.54^o^	2.10^q^
	375	8.21^f^	2.53^jk^	6.58^no^	2.11^pq^
Cold storage (days)					

10	0	8.42^ef^	2.56^ij^	8.99^d^	2.67^f^
	125	8.59^e^	2.61^g^	6.88^lm^	2.13^o-q^
	250	8.61^e^	2.62^g^	6.87^mn^	2.14^op^
	375	8.62^e^	2.62^g^	6.87^mn^	2.15^o^

20	0	9.00^d^	2.69^f^	9.68^b^	2.80^ab^
	125	9.00^d^	2.70^ef^	7.21^i-k^	2.23^n^
	250	9.25^cd^	2.71^d-f^	7.20^jk^	2.23^n^
	375	9.00^d^	2.70^ef^	7.17^kl^	2.24^n^

30	0	9.37^c^	2.78^bc^	11.26^a^	2.81^a^
	125	9.32^c^	2.73^de^	7.51^h^	2.30^m^
	250	9.37^c^	2.74^d^	7.50^hi^	2.31^m^
	375	9.37^c^	2.75^cd^	7.49^h-j^	2.32^m^

Overall mean	Topping	8.75	2.63	7.68	2.33
	Ambient	8.05	2.48	7.06	2.23
	Cold storage	8.99	2.68	7.88	2.36
	Nonsoaked	8.67	2.61	9.61	2.72
	GA_3_ soaked	8.78	2.64	7.03	2.20

Means with different letter(s) in columns have significant differences according to DMRT at 5% probability level, and CN = clove number and ACW = average clove weight.

**Table 7:** Bulb dry matter, total dry biomass, and total bulb yield of garlic cloves as influenced by the interaction effects of clove topping, cold storage duration, and gibberellic acid.

Storage	Topping	Whole clove			Topped clove		
	GA_3_ (mg/l)	BDM (%)	TDB (g)	TBY (t/ha)	BDM (%)	TDB (g)	TBY (t/ha)
Ambient temperature	0	27.77^j^	9.74^k^	8.02^h^	30.17^h^	12.34^h^	9.43^f^
	125	27.95^j^	10.06^k^	8.17^h^	23.25^n^	5.19^o^	4.73^l^
	250	28.94^i^	11.14^j^	8.80^g^	23.10^n^	5.12^o^	4.70^l^
	375	28.93^i^	11.15^j^	8.80^g^	23.12^n^	5.16^o^	4.72^l^
Cold storage (days)							

10	0	29.75^h^	11.76^i^	9.07^g^	31.36^g^	13.92^g^	10.22^e^
	125	30.16^h^	12.39^h^	9.45^f^	24.28^m^	6.28^n^	5.52^k^
	250	30.22^h^	12.46^h^	9.48^f^	24.27^m^	6.29^n^	5.53^k^
	375	30.24^h^	12.49^h^	9.49^f^	24.36^m^	6.32^n^	5.53^k^

20	0	31.39^g^	13.99^g^	10.27^e^	34.17^bc^	16.95^bc^	11.53^b^
	125	31.43^g^	14.01^g^	10.28^e^	25.58^l^	7.32^m^	6.23^j^
	250	32.15^f^	14.78^f^	10.63^d^	25.60^l^	7.33^m^	6.24^j^
	375	31.37^g^	14.00^g^	10.28^e^	25.68^l^	7.33^m^	6.23^j^

30	0	33.59^cd^	16.11^cd^	11.25^bc^	37.49^a^	21.29^a^	13.66^a^
	125	32.96^e^	15.53^e^	10.96^cd^	26.68^k^	8.34^l^	6.91^i^
	250	32.95^e^	15.58^e^	10.98^c^	26.76^k^	8.37^l^	6.90^i^
	375	32.98^de^	15.61^e^	10.99^c^	26.72^k^	8.38^l^	6.92^i^

Overall mean	Topping	30.80	13.17	9.81	27.04	9.12	7.19
	Ambient	28.40	10.52	8.45	24.91	6.95	5.89
	Cold storage	31.60	14.06	10.26	27.74	9.84	7.62
	Nonsoaked	30.62	12.90	9.65	33.30	16.12	11.21
	GA_3_ soaked	30.86	13.27	9.86	24.95	6.78	5.85

Means with different letter(s) in columns have significant differences according to DMRT at 5% probability level, and BDM = bulb dry matter, TDB = total dry biomass, and TBY = total bulb yield.

**Table 8:** Mean square for the effect of clove topping (CT), cold storage duration (DLTS), gibberellic acid (GA_3_), and season on clove size category of garlic (in number and weight, respectively).

Source of variation	Small	Medium	Large	Very large	Small	Medium	Large	Very large
Rep (2)	0.1350^*∗∗*^	0.3522^*∗∗*^	1.4701^*∗∗*^	0.1509^*∗∗*^	0.4215^*∗∗*^	1.2288^*∗∗*^	7.6652^*∗∗*^	5.7791^*∗∗*^
Sn (1)	0.6510^*∗∗*^	1.3665^*∗∗*^	4.1475^*∗∗*^	1.8113^*∗∗*^	3.2199^*∗∗*^	5.0375^*∗∗*^	19.9085^*∗∗*^	11.8686^*∗∗*^
CT (1)	26.7333^*∗∗*^	13.3528^*∗∗*^	20.8565^*∗∗*^	4.4417^*∗∗*^	54.2078^*∗∗*^	42.3494^*∗∗*^	115.6433^*∗∗*^	224.4736^*∗∗*^
DLTS (3)	12.2251^*∗∗*^	7.7626^*∗∗*^	14.8367^*∗∗*^	3.5035^*∗∗*^	25.3350^*∗∗*^	24.8293^*∗∗*^	82.9468^*∗∗*^	122.0499^*∗∗*^
GA_3_ (4)	10.7194^*∗∗*^	7.5301^*∗∗*^	13.0730^*∗∗*^	2.6423^*∗∗*^	22.0579^*∗∗*^	24.0765^*∗∗*^	72.7908^*∗∗*^	122.6774^*∗∗*^
CT *×* DLTS (3)	0.1174^*∗∗*^	0.0828^*∗∗*^	0.3055^*∗∗*^	0.2176^*∗∗*^	0.2367^*∗∗*^	0.2523^*∗∗*^	1.6442^*∗∗*^	1.1200^*∗∗*^
CT *×* GA_3_ (4)	11.9894^*∗∗*^	8.6413^*∗∗*^	15.5421^*∗∗*^	2.9960^*∗∗*^	24.7344^*∗∗*^	27.6949^*∗∗*^	87.0224^*∗∗*^	140.8317^*∗∗*^
DLTS *×* GA_3_ (12)	0.0670^*∗∗*^	0.1688^*∗∗*^	0.2553^*∗∗*^	0.0430^*∗∗*^	0.1426^*∗∗*^	0.5481^*∗∗*^	1.4120^*∗∗*^	2.6263^*∗∗*^
Sn *×* CT (1)	0.0010^ns^	0.0105^ns^	0.0555^ns^	0.0292^*∗∗*^	0.1516^*∗*^	0.0921^ns^	0.5805^*∗*^	0.1116^ns^
Sn *×* DLTS (3)	0.00004^ns^	0.0102^ns^	0.0445^ns^	0.0047^ns^	0.0306^ns^	0.0537^ns^	0.3960^*∗*^	0.4609^*∗*^
Sn *×* GA_3_ (4)	0.0004^ns^	0.0087^ns^	0.0361^ns^	0.0038^ns^	0.0377^ns^	0.0462^ns^	0.3324^*∗*^	0.1948^ns^
CT *×* DLTS *×* GA_3_ (12)	0.0827^*∗∗*^	0.0399^*∗∗*^	0.0809^*∗∗*^	0.0317^*∗∗*^	0.1622^*∗∗*^	0.1302^*∗∗*^	0.4484^*∗∗*^	0.6622^*∗∗*^
Sn *×* DLTS *×* GA_3_ (12)	0.0001^ns^	0.0003^ns^	0.0011^ns^	0.0001^ns^	0.0005^ns^	0.0014^ns^	0.0096^ns^	0.0155^ns^
Sn *×* CT *×* DLTS (3)	0.00004^ns^	0.0009^ns^	0.0003^ns^	0.0003^ns^	0.0004^ns^	0.0051^ns^	0.0065^ns^	0.0504^ns^
Sn *×* CT *×* GA_3_ (4)	0.0004^ns^	0.0098^ns^	0.0491^ns^	0.0069^ns^	0.0389^ns^	0.0537^ns^	0.4248^*∗∗*^	0.2375^ns^
Sn *×* CT *×* DLTS *×* GA_3_ (12)	0.0001^ns^	0.0002^ns^	0.0002^ns^	0.0002^ns^	0.0016^ns^	0.0006^ns^	0.0024^ns^	0.0035^ns^
Error (158)	0.0173	0.0088	0.0206	0.0032	0.0347	0.0263	0.1117	0.1288
CV (%)	9.11	6.88	3.65	3.39	9.14	6.78	3.62	4.6

ns, ^*∗*^and ^*∗∗*^nonsignificant, significant at *P* < 0.05 and *P* < 0.01, respectively.

**Table 9:** Interaction effects of cold storage duration, clove topping, and gibberellic acid on clove size category based on number.

Storage	Topping	Whole clove				Topped clove			
	GA_3_ (mg/l)	Small	Medium	Large	Very large	Small	Medium	Large	Very large
Ambient	0	1.72^e^	1.04^j^	3.59^i^	1.54^h^	1.18^g^	1.49^gh^	4.10^fg^	1.76^f^
	125	1.72^e^	1.04^j^	3.59^i^	1.54^h^	3.23^a^	0.28^n^	2.35^m^	0.74^l^
	250	1.45^f^	1.27^i^	3.84^h^	1.65^g^	3.19^a^	0.26^n^	2.35^m^	0.74^l^
	375	1.45^f^	1.27^i^	3.84^h^	1.65^g^	3.24^a^	0.26^n^	2.35^m^	0.73^l^
Cold storage (days)									

10	0	1.38^f^	1.39^h^	3.94^gh^	1.71^fg^	0.95^h^	1.74^f^	4.41^e^	1.89^de^
	125	1.18^g^	1.52^g^	4.12^f^	1.76^f^	2.52^b^	0.45^m^	2.70^l^	1.22^k^
	250	1.18^g^	1.52^g^	4.15^f^	1.76^f^	2.51^b^	0.45^m^	2.70^l^	1.22^k^
	375	1.18^g^	1.52^g^	4.15^f^	1.76^f^	2.51^b^	0.45^m^	2.70^l^	1.22^k^

20	0	0.95^h^	1.77^ef^	4.41^e^	1.87^e^	0.47^j^	2.19^b^	4.93^b^	2.09^b^
	125	0.95^h^	1.77^ef^	4.41^e^	1.87^e^	2.25^c^	0.62^l^	3.02^k^	1.32^j^
	250	0.88^h^	1.87^de^	4.57^de^	1.93^d^	2.24^c^	0.62^l^	3.02^k^	1.32^j^
	375	0.95^h^	1.77^ef^	4.41^e^	1.87^e^	2.24^c^	0.62^l^	2.99^k^	1.32^j^

30	0	0.52^j^	2.07^c^	4.76^c^	2.03^bc^	0.25^k^	2.65^a^	5.87^a^	2.49^a^
	125	0.72^i^	1.94^d^	4.67^cd^	2.00^c^	1.98^d^	0.78^k^	3.31^j^	1.43^i^
	250	0.72^i^	1.97^cd^	4.69^cd^	2.00^c^	1.97^d^	0.78^k^	3.31^j^	1.43^i^
	375	0.72^i^	1.97^cd^	4.69^cd^	2.00^c^	1.97^d^	0.78^k^	3.30^j^	1.43^i^

Overall mean	Topping	1.10	1.61	4.24	1.81	2.04	0.90	3.34	1.40
	Ambient	1.58	1.15	3.71	1.60	2.71	0.57	2.79	0.99
	Cold storage	0.94	1.76	4.41	1.88	1.82	1.01	3.52	1.53
	Nonsoaked	1.14	1.57	4.17	1.79	0.71	2.02	4.83	2.06
	GA_3_ soaked	1.09	1.62	4.26	1.81	2.49	0.53	2.84	1.18

Means with different letter(s) in columns have significant differences according to DMRT at 5% probability level.

**Table 10:** Interaction effects of cold storage duration, clove topping, and gibberellic acid on clove size category based on weight (g).

Storage	Topping	Whole clove				Topped clove			
	GA3(mg/l)	Small	Medium	Large	Very large	Small	Medium	Large	Very large
Ambient	0	2.43^e^	1.80^k^	8.40^k^	6.56^j^	1.67^g^	2.61^hi^	9.61^g-i^	8.30^gh^
	125	2.43^e^	1.82^k^	8.41^k^	6.60^j^	4.59^a^	0.45^o^	5.47^o^	3.43^n^
	250	2.05^f^	2.21^j^	8.99^j^	7.44^i^	4.54^a^	0.41^o^	5.48^o^	3.32^n^
	375	2.05^f^	2.22^j^	9.00^j^	7.46^i^	4.60^a^	0.42^o^	5.49^o^	3.34^n^
Cold storage (days)									

10	0	1.95^f^	2.44^i^	9.23^ij^	7.94^h^	1.33^h^	3.05^g^	10.33^f^	9.32^f^
	125	1.67^g^	2.67^h^	9.67^g^	8.42^g^	3.57^b^	0.75^n^	6.30^n^	4.09^m^
	250	1.67^g^	2.68^h^	9.73^g^	8.45^g^	3.56^b^	0.76^n^	6.31^n^	4.11^m^
	375	1.67^g^	2.69^h^	9.74^g^	8.47^g^	3.56^b^	0.77^n^	6.32^n^	4.13^m^

20	0	1.33^h^	3.11^fg^	10.33^f^	9.44^ef^	0.63^j^	3.87^b^	11.61^b^	11.10^b^
	125	1.33^h^	3.13^fg^	10.35^ef^	9.47^ef^	3.19^c^	1.04^m^	7.05^m^	4.75^l^
	250	1.20^hi^	3.28^ef^	10.72^de^	9.85^de^	3.18^c^	1.05^m^	7.06^m^	4.77^l^
	375	1.33^h^	3.12^fg^	10.35^ef^	9.46^ef^	3.18^c^	1.06^m^	7.01^m^	4.78^l^

30	0	0.70^j^	3.64^c^	11.16^c^	10.59^c^	0.30^k^	4.70^a^	13.78^a^	12.95^a^
	125	0.99^i^	3.41^de^	10.95^cd^	10.09^d^	2.81^d^	1.34^l^	7.74^l^	5.41^k^
	250	0.99^i^	3.46^c-e^	11.02^cd^	10.23^cd^	2.80^d^	1.35^l^	7.75^l^	5.43^k^
	375	0.99^i^	3.47^cd^	11.03^cd^	10.25^cd^	2.80^d^	1.36^l^	7.73^l^	5.44^k^

Overall mean	Topping	1.55	2.82	9.94	8.80	2.89	1.56	7.81	5.92
	Ambient	2.24	2.01	8.70	7.01	3.85	0.97	6.51	4.60
	Cold storage	1.32	3.09	10.36	9.39	2.57	1.76	8.25	6.36
	Nonsoaked	1.60	2.75	9.78	8.63	0.98	3.56	11.33	10.42
	GA_3_ soaked	1.53	2.85	9.99	8.85	3.53	0.89	6.64	4.42

Means with different letter(s) in columns have significant differences according to DMRT at 5% probability level.

## Data Availability

The data used to support the findings of this study are included within the article.

## References

[1] Desta B., Tena N., Amare G. (2021). Growth and bulb yield of garlic as influenced by clove size. *The Scientific World Journal*.

[2] Takagi H., Rabinowitch H. D., Brewster J. L. (1990). Garlic *Allium sativum* L.. *Onions and Allied crops, (Biochemistry, Food Science, and Minor Crops)*.

[3] Del Pozo A., Gonzalez G. (2005). Developmental responses of garlic to temperature and photoperiod. *Agricultural Technology*.

[4] Bhuiya M. A. K., Rahim M. A., Chowdhury M. N. A. (2003). Effect of planting time, mulch and irrigation on the growth and yield of garlic. *Asian Journal of Plant Sciences*.

[5] Ade-Ademilua O. E., Iwaotan T. O., Osaji T. C. (2009). Pre-Planting (cold) treatments of *Allium sativum* cloves improves its growth and yield under open field and open shade conditions. *Journal of Plant Sciences*.

[6] Siddique M. A., Rabbani M. G. (1985). Growth and bulbing of garlic in response to low temperature treatment of bulb and planting date. *Bangladesh Journal of Botany*.

[7] Rizk F. A., El-Habbasha K. M. (1996). Flowering and seed yield of onion (*Allium cepa* L.) plants as affected by dates of planting and some growth regulators. *Journal of Horticulture*.

[8] Chowdhury A. K. (2003). Effect of plant growth regulators on growth physiological characters and yield attributes of barley.

[9] Amal M. E., Hegazi A. M. (2009). Effect of acetylsalicylic acid, indole-3- bytric acid and gibberellic acid on plant growth and yield of pea (*Pisum sativum* L.). *Australian Journal of Basic and Applied Sciences*.

[10] Moon W., Lee B. Y. (1980). Influence of short day treatment on the growth and levels of endogenous growth substances in garlic (*Allium sativum* L.) plants. *Journal of the Korean Society for Horticultural Science*.

[11] Ouzounidou G., Giannakoula A., Asfiiand M., Ilias I. (2011). Differential response of onion and garlic against plant growth regulator. *Pakistan Journal of Botany*.

[12] Arifin N. S., Okubo H., Miho N. (1999). Dormancy in Shallot *(Allium cepa* var. *ascalonicum)* and *Allium X wakegi* bulbs and its breaking by scale cutting. *Journal of the Faculty of Agriculture, Kyushu University*.

[13] Peter K. V. (2006). *Handbook of Herbs and Spices, Volume 3*.

[14] Yamazaki H., Ishida N., Katsura N., Kano H., Nishijima T., Koshioka M. (1995). Changes in carbohydrate composition and water status during bulb development of *Allium wakegi* Araki. *Bulletin of National Research Institute of Vegetables, Ornamental Plants and Tea*.

[15] Tabour G., Zelleke A. (2000). *Achievements in Shallot and Garlic Research Report*.

[16] Gedamu F. (2005). Effects of clove size and plant density on the bulb yield and yield components of garlic (Allium sativum L.) in Awabel Woreda, Eastern Gojjam Zone.

[17] Gomez K. A., Gomez A. A. (1984). *Statistical Procedures of Agricultural Research*.

[18] Lin P. C., Roberts A. N. (1970). Scale function in growth and flowering of *Lilium longiflorum*, Thunb. “Nellie white”. *Journal of the American Society for Horticultural Science*.

[19] Wang S. Y., Roberts A. N. (1970). Physiology of dormancy in *Lilium longiflorum* “ace,” thunb. *Journal of the American Society for Horticultural Science*.

[20] Yamazaki H., Nishijima T., Koshioka M., Miura H. (2002). Gibberellins do not act against abscisic acid in the regulation of bulb dormancy of *Allium wakegi* Araki. *Plant Growth Regulation*.

[21] Teaster N. D., Motes C. M., Tang Y., Wiant W. C., Cotter M. Q. (2007). N-Acylethanolamine metabolism interacts with absicisic acid signaling in *Arabidopsis thaliana* seedlings. *The Plant Cell Online*.

[22] Solomina V. F. (1977). Changes in the activity of endogenous cytokinins during the relative dormancy of garlic bulbs. *Horticultural Abstracts*.

[23] Silva N. F., Casali V. W. D. (1987). Effects of low temperature storage and planting date on dormancy in the garlic cultivar *Peruano*. *Horticultural Abstracts*.

[24] Langens-Gerrits M. M., Miller W. B. M., Croes A. F., De Klerk G. J. (2003). Effect of low temperature on dormancy breaking and growth after planting in lily bulblets regenerated *in vitro*. *Plant Growth Regulation*.

[25] Satin S. M. E., Lopez M. (1994). Effects of storage temperature on growth and bulb formation in four garlic (*Allium sativum* L.) cultivars. *Pakistan Journal of Botany*.

[26] Khokhar K. M., Hadley P., Pearson S. (2007). Effect of cold temperature durations of onion sets in store on the incidence of bolting, bulbing and seed yield. *Scientia Horticulturae*.

[27] Aura K. (1968). Studies on the vegetatively propagated onions cultivated in Finnland, with special reference to flowering and storage. IX. The influence of various storage temperatures on flowering and yield in the North Finnish onion strain. *Annales Agriculturae Fenniae*.

[28] Butt A. M. (1968). Vegetative growth, morphogenesis and carbohydrate content of the onion plant as a function of light and temperature under field and controlled conditions. *Mededelingen Landbouwhogeschool Wageningen*.

[29] Palilov N. A. (1969). The biological bases of onion storage. *Proceedings of the Symposium of vegetable storage, Acta Horticulturae*.

[30] Rahim M. A., Fordham R. (2001). Environmental manipulation for controlling bulbing in garlic. *Acta Horticulturae*.

[31] Cantwell M. I., Kang J., Hong G. (2003). Heat treatments control sprouting and rooting of garlic cloves. *Postharvest Biology and Technology*.

[32] Vazquez B. M., Lopez E. G., Mercado S. E., Castano T. E., Leon V. G., Ratindo K. E. (2006). Low temperature storage of garlic for spring planting. *Horticultural Science*.

[33] Rahman M. H., Haque M. S., Karim M. A., Ahmed A. (2006). Effects of Gibberellic acid on breaking dormancy in garlic (*Allium sativum* L.). *International Journal of Agriculture and Biology*.

[34] Daykin A., Scott I. M., Francis D., Causton D. R. (1997). Effects of gibberellin on the cellular dynamics of dwarf pea internode development. *Planta*.

[35] Hisamatsu T., Koshioka M., Kubota S., King R. W. (1998). Effect of gibberellin A_4_ and Gibberellic acid biosynthesis inhibitors on growth and flowering of stock (*Matthiola incana* (L.) R. Br.). *Journal of the Japanese Society for Horticultural Science*.

[36] Petric M., Jevremovic S., Trifunovic M. (2013). The effect of low temperature and GA_3_ treatments on dormancy breaking and activity of antioxidant enzymes in *Fritillaria meleagris* bulblets cultured *in vitro*. *Acta Physiologiae Plantarum*.

[37] Rahman S. M., Islam A. M., Haque S. M., Karim A. M. (2004). Effects of planting date and Gibberellic acid on the growth and yield of garlic (*Allium sativum L*). *Asian Journal of Plant Sciences*.

[38] Watts S., Rodriguez J. L., Evans S. E., Davies W. J. (1981). Root and shoot growth of plants treated with abscisic acid. *Annals of Botany*.

[39] Munns R., Cramer G. R. (1996). Is coordination of leaf and root growth mediated by abscisic acid? Opinion. *Plant and Soil: International journal on plant-soil relationships*.

[40] Dutcher R. D., Powell L. E. (1972). Culture of apple shoots from buds *in vitro*. *Journal of the American Society for Horticultural Science*.

[41] Singha S., Powell L. E. (1978). Response of apple buds cultured *in vitro* to abscisic acid. *Journal of the American Society for Horticultural Science*.

[42] Lucidos J. G., Younis A., Hwang Y. J., Son B. G., Lim K. B. (2014). Determination ofoptimum conditions for breaking bulb dormancy in relation to growth and flowering in *Lilium hansonii*. *Horticulture Environment and Biotechnology*.

[43] Kurtar E. S., Ayan A. K. (2005). Effects of gibberellic acid (GA_3_) and indole acetic acid (IAA) on flowering, stalk elongation and bulb characteristics of tulip (*Tulipa gesneriana* var. Cassini). *Pakistan Journal of Biological Sciences*.

[44] Park Y. B., Lee Y. B. (1989). The effect of seed bulb storage temperature and storage period on the carbohydrate and endogenous hormone contents of Northern and Southern ecotypes of garlic in Cheju. *Horticultural Abstracts*.

[45] Moustafa Y. M. (2011). Performance of new imported foreign garlic genotypes grown under the Egyptian conditions. *Egyptian Journal of Agricultural Research*.

[46] Youssef S. N. (2013). Growth and bulbing of garlic as influenced by low temperature and storage period treatments. *Journal of Rural Observation*.

[47] Qaryouti M. M., Kasrawim M. A. (1995). Storage temperature of seed bulbs and planting date influence on garlic I. Emergence, vegetative growth, bulbing and maturity. *Advanced Horticultural Science*.

[48] Rahim M. A., Fordam R. (1994). Control of bulbing in garlic. *Acta Horticulturae*.

[49] De Klerk G. J. (2009). A cold treatment promotes both sprouting and sink strength of lily bulblets. *Propagation of Ornamental Plants*.

[50] Kamenetsky R., Shafir I. L., Zemah H., Barzila A., Rabinowitch H. D. (2004). Environmental control of garlic growth and florogenesis. *Journal of the American Society for Horticultural Science*.

[51] Brewster J. L. (2008). *Onions and Other Vegetable Alliums (2^nd^edn*.

[52] Bandara M. S., Krieger K., Slinkard A. E., Tanino K. K. (2000). Pre-plant chilling requirements for cloving of spring planted garlic. *Journal of Plant Sciences*.

[53] Ferreira F. A., Cheng S. S., Cardoso M. R. O. (1980). Effects of pre-planting on the vegetative cycle, production, chemical composition and post-harvest conservation of garlic, Chonan cultivar, aiming to produce off-season at elevated altitude of 1300 m. *Journal Olericultura*.

[54] Pyo H. K., Lee B. Y., Moon W., Woo J. K. (1979). Study on the development of a new cultural system for garlic. The effect of low-temperature treatment of seed bulbs, light interruption and supplemental lighting on the growth and bulbing of garlic in plastic film house. *Journal of the Korean Society for Horticultural Science*.

[55] Biasi J., Mueller S. (1984). Influence of the immersion of garlic in water. *Brazilian Congress of Oliculture, 24*.

[56] Manjula S., Bandara K., Krieger A. E., Slinkard K., Tanino K. (2000). Preplant chilling requirements for cloving of spring planted garlic. *Canadian Journal of Plant Science*.

